# Do Members of Disadvantaged Groups Explain Group Status With Group Stereotypes?

**DOI:** 10.3389/fpsyg.2021.750606

**Published:** 2021-11-18

**Authors:** Juliane Degner, Joelle-Cathrin Floether, Iniobong Essien

**Affiliations:** ^1^Department of Psychology, Universität Hamburg, Hamburg, Germany; ^2^Department of Social and Organisational Psychology of Social Work, Leuphana University of Lüneburg, Lüneburg, Germany

**Keywords:** disadvantaged groups, system justification theory, rejection identification model, intergroup attitudes, status perceptions

## Abstract

Recent research on group attitudes in members of disadvantaged groups has provided evidence that group evaluations closely align with societal stigma, reflecting outgroup favoritism in members of those groups that are most strongly stigmatized. While outgroup favoritism is clearly evident among some groups, there is still debate about the psychological mechanisms underlying outgroup favoritism. The current research focuses on a less intensively examined aspect of outgroup favoritism, namely the use of status-legitimizing group stereotypes. We present data from members of four disadvantaged groups (i.e., persons who self-categorize as gay or lesbian, *n* = 205; Black or African American, *n* = 209; overweight *n* = 200, or are aged 60–75 years *n* = 205), who reported the perceived status of their ingroup and a comparison majority outgroup and provided explanations for their status perceptions. Contrary to assumptions from System Justification Theory, participants rarely explained perceived group status differences with group stereotypes, whereas they frequently explained ingroup disadvantage with perceived stigmatization and/or systemic reasons. Further exploratory analyses indicated that participants’ status explanations were related to measures of intergroup attitudes, ideological beliefs, stigma consciousness, and experienced discrimination. Our results highlight the need to develop a better understanding whether, under what circumstances, and with which consequences members of disadvantaged groups use group stereotypes as attributions of ingroup status and status differences.

## Introduction

People frequently use attributions—explanations for positive or negative life events and outcomes—to navigate their social worlds (e.g., Heider, 1958; Malle, 2011). Besides creating a sense of understanding, attributions serve further psychological needs, such as desires for meaning and purpose, control and mastery, self-worth and distinctiveness (e.g., Baumeister, 1991). Life outcomes are not only affected by individual behaviors, vices, and virtues, but also by peoples’ social group memberships and their embeddedness in social systems and hierarchies. Especially for members of disadvantaged or stigmatized groups, it seems paramount to make sense of the social conditions and constraints that govern their lives. For example, attributing low societal status of one’s ingroup to internal characteristics such as group members’ abilities or motivations may have entirely different implications for one’s own aspirations and behaviors than attributing lower societal status to systemic inequality or ingroup disadvantage (Dupree et al., 2021).

The current research explores the reasons members of various disadvantaged groups provide when asked to explain the perceived status of their ingroup and whether and to what extent various attributions are associated with evaluations of ingroups and outgroups. This research was mainly stimulated by critical reflections on theoretical assumptions of System Justification Theory (SJT, Jost and Banaji, 1994; Jost, 2020) and was further informed by the Rejection-Identification Model (RIM, Branscombe et al., 1999; Schmitt and Branscombe, 2002). Both theories contain opposing assumptions regarding how members of disadvantaged groups make sense of perceived own group status.

### System Justification Theory

The general premise of SJT is that peoples’ thinking, feeling, and behavior is influenced by a principal system justification motive as a higher-order psychological need: a motivated desire to perceive social systems in which people are embedded as fair, legitimate, and justifiable (Jost and Banaji, 1994). Applied to perceptions of group status, a major consequence of this motivated perception of social systems is that people attribute objective or perceived disparities between social groups to internal causes rather than to contextual or systemic causes (Jost, 2020). Due to these internal group-focused attributions, beliefs about advantaged groups are presumed to include stereotypic characteristics perceived to cause their relative success (e.g., higher ability or effort) and beliefs about disadvantaged groups are presumed to include stereotypic characteristics perceived to cause their relative failure (e.g., lower ability or effort). SJT further presumes that this group-focused internal attribution style makes social systems and their status hierarchies appear fair, just, and legitimate, because it bolsters the belief that people get what they deserve and deserve what they get (cf. Lerner, 1980). The theory also postulates that system motivation tendencies result in the evaluative preferences for higher status advantaged groups, thus ingroup preference in individuals who are members of these advantaged groups and outgroup preference in individuals who are members of disadvantaged groups.

Although SJT includes assumptions about individual and contextual variations affecting the strength of system-justifying motivations (see Jost, 2020, for a recent overview), its general premise is that system-justifying motivation are shared by both members of advantaged and disadvantaged groups – thus by those who benefit from and those who are harmed by the status quo. Consequently, SJT postulates that both, members of advantaged and disadvantaged groups, employ the same system-justifying strategies and thus endorse similar group stereotypes that explain, rationalize and justify group disparities in society (e.g., in terms of wealth, educational outcomes, health, or representation). In line with this notion, SJT presumes that in members of disadvantaged groups, system-justifying motives result in “internalized inferiority” (Jost et al., 2002, 2004; Mentovich and Jost, 2008; Jost and van der Toorn, 2012)—the holding of beliefs, which are harmful to the self or one’s ingroup. Thus, instead of attributing one’s own disadvantage or one’s ingroup status to societal conditions or situational constraints (e.g., inequality, discrimination), members of disadvantaged groups are presumed to explain perceived status differences by sharing society’s stereotypic beliefs about ingroup and outgroup characteristics. SJT further postulates that holding such system-justification beliefs leads to internalized, devaluation of the ingroup, eventually resulting in outgroup favoritism – an evaluative preference for higher status outgroups over the ingroup.

There are ample empirical findings that appear to support SJT’s assumptions about group evaluations (Jost et al., 2002; Livingston, 2002; Nosek et al., 2002; Rudman et al., 2002; Ashburn-Nardo et al., 2003). For example, recent meta-analytical findings suggest that intergroup evaluations in members of disadvantaged groups closely align with societal stigmatization: Disadvantaged groups were more likely to display outgroup favoritism to the extent that their ingroup was negatively evaluated by others in society (Essien et al., 2021). Note, however, that this meta-analysis also reported high levels of heterogeneity and systematic differences between groups: Whereas members of the most negatively evaluated disadvantaged groups exhibited outgroup favoritism, members of less negatively evaluated disadvantaged groups exhibited ingroup favoritism.

There are far fewer empirical findings available on the use of stereotypes by members of disadvantaged groups, and as we discuss below, they provide only limited support for SJT’s postulate that members of disadvantaged groups use societally shared group stereotypes as explanations for perceived status differences between groups, thus legitimizing and justifying their own group’s disadvantage (e.g., Jost and Banaji, 1994). Furthermore, claims about the use of group stereotypes by members of disadvantaged groups are challenged by assumptions embedded in the Rejection Identification Model, as we discuss next.

### Assumptions From the Rejection-Identification-Model

The RIM (Branscombe et al., 1999; Schmitt and Branscombe, 2002) proposes that members of disadvantaged groups not only recognize individual and societal prejudice and discrimination as causes of their disadvantage, but also actively use these as attributions of negative interaction experiences and outcomes (Branscombe et al., 1999; Schmitt and Branscombe, 2002; see also Crocker and Major, 1989). The RIM further proposes that the extent to which members of disadvantaged groups attribute negative experiences to group-based discrimination has important implications for psychological well-being and social identity processes—an assumption widely supported by empirical research (e.g., meta-analysis by Schmitt et al., 2014). Specifically, the RIM proposes that perceiving discrimination can actually support coping with stigma, protect well-being and self-esteem, and strengthen affective ties with the ingroup (i.e., ingroup identification), especially when societal treatment of the ingroup or the self is construed as unjustified and illegitimate. A large body of experimental and correlational research conducted with members of disadvantaged groups has supported the presumed relationships between perceived discrimination, well-being, and social identification (e.g., Giamo et al., 2012; Ramos et al., 2012; Curll and Brown, 2020; Mazzoni et al., 2020). Most importantly for the current research, this literature documents that members of disadvantaged groups often show a willingness to attribute various negative life experiences to pervasive discrimination, and that this tendency is associated with substantial interindividual variation and variation between different groups.

### Comparing Theoretical Perspectives and Developing an Empirical Perspective

The apparent opposition between these two theoretical approaches and their supporting empirical findings is acknowledged in SJT. For example, a recent formulation of SJT includes the statement that “few observers would argue that African Americans or other racial or ethnic minorities *explicitly* endorse the legitimacy of racial inequality” (Jost, 2020, p. 119, emphasis added). SJT, however, disputes that open expressions of perceived discrimination and stigma consciousness by members of disadvantaged groups reflect genuine personal beliefs. Instead, such responses are interpreted as reflecting mere conformity to social norms pressuring people to exaggerate self- and group-interested responses—especially in groups that “are known to have been historical targets of discrimination and prejudice” (Jost, 2020, p. 118). When it comes to *personal beliefs*, however, SJT presumes that members of disadvantaged groups often do not acknowledge discrimination as a cause of their disadvantage but instead internalize negative stigmatization and attribute their own group status to internal group characteristics (i.e., stereotypes). However, because of presumed normative pressures, neither group stereotype endorsement nor outgroup favoritism are openly expressed. Given that research supporting the RIM mostly used measures that offer pre-formulated attributions to stigmatization and discrimination (e.g., stigma consciousness scale, Pinel, 1999), rather than investigating participants’ own responses, one may indeed argue that the measurement process itself imposes normative pressure, triggering acquiescence biases that lead to an overestimation of agreement with perceived stigmatization attributions. In order to better understand whether and to what extent members of disadvantaged groups hold personal beliefs containing group stereotypes as explanations and/or justifications for group status, SJT proposes to rely on either open-ended, non-reactive, qualitative measures (Hypothesis H6a, Jost et al., 2004; Jost, 2020) or implicit, indirect, and unobtrusive measures (Hypothesis H6b, Jost et al., 2004; Jost, 2020) because both are assumed to be less likely affected by social desirability concerns and perceived normative pressures. Hypothesis 6b has received tremendous attention in the implicit cognition literature with a large number of studies investigating ingroup and outgroup favoritism among members of disadvantaged groups (e.g., Essien et al., 2021). Hypothesis 6a, on the other hand, has rarely been investigated. Although there is a vast body of research demonstrating that ingroup stereotyping in members of disadvantaged groups sometimes align with societal stereotyping (e.g., mainly in research on gender role stereotyping), only few studies have directly investigated SJT’s postulate that members of disadvantaged groups use societally shared group stereotypes as explanations of perceived status differences between groups (e.g., Jost and Banaji, 1994). To our knowledge, the only available empirical test of these assumptions stems from three experiments conducted by Jost and Burgess (2000) and Jost (2001). They manipulated perceived ingroup status as relatively high vs. low compared to another outgroup and assessed measures of group stereotyping. Specifically, university students were provided bogus information about average financial incomes, career advancement, and educational outcomes of graduates of their own versus another university (i.e., Yale vs. Stanford; Virginia vs. Maryland; U.C.S.P. vs. U.C.L.A.), such that participants perceived their ingroup as relatively higher or lower in socio-economic success than the outgroup. In one study, the Yale (vs. Stanford) study, participants also provided explanations for the perceived status differences between the two groups (Jost, 2001). Participants’ open-ended responses were coded for the use of group stereotypes, by counting favorable, unfavorable, or neutral expressions about either the ingroup or the outgroup.^1^

Results indeed indicated a pattern of ingroup favoritism in the higher-status condition, with more favorable ingroup characterizations and more unfavorable outgroup characterizations, and a reversed pattern in the lower-status condition, with more unfavorable ingroup characterizations and more favorable outgroup characterizations. From these observations (and consistent patterns of trait ratings in the two other studies, see Jost and Burgess, 2000) it was concluded that “*low-status group members do not attribute their inferior position to situational factors or extenuating circumstances, but rather seem to internalize the inequality in the form of internal attributions about unfavorable characteristics of the ingroup and favorable characteristics of the outgroup*” (Jost, 2001, p. 97). While these experiments provide interesting and important insights about effects of perceived status differences on ingroup and outgroup characterizations, they leave several important questions unanswered. For example, participants in these studies were students at elite institutions, posing the question whether findings generalize to life-long experiences of members of disadvantaged groups. Moreover, participants’ responses were, to our knowledge, only coded with regard to group characterizations, but not with regard to other (e.g., situational, systemic) attributions. It is thus an open question whether members of groups who face real-world disadvantages similarly attribute their own or their ingroup’s disadvantage to negative ingroup stereotypes.

We addressed these limitations by asking members of disadvantaged groups to provide explanations of perceived group status and status differences. We used the same open-ended methodology reported by Jost (2001) and coded participants’ responses. Our aim was to quantify (a) occurrences of stereotypical ingroup or outgroup characterizations as expected in SJT and, (b) occurrences of perceived discrimination as expected in the RIM. Furthermore, we explored (c) whether participants’ responses contained attributions to systemic factors, such as institutionalized disadvantages and group discrimination.

### The Current Research

The current research aimed at complementing previous experimental research (i.e., Jost and Burgess, 2000; Jost, 2001) by studying members of four real-life disadvantaged groups: Individuals who self-categorized as gay or lesbian, as Black or African American, as overweight and individuals aged 60–75 years. Instead of manipulating status perceptions, we asked participants first to indicate the perceived status of their respective ingroup and the contrasting advantaged outgroup and then asked them to provide explanations for their status responses in an open-ended, non-reactive, qualitative measure, highly similar to the one used by Jost (2001). Similar to Jost, we coded responses with regard to the occurrence of favorable or unfavorable characterizations of the ingroup and the outgroup. Extending the coding procedure, we additionally coded whether stereotypic group characterizations indicated participants’ stereotype endorsement—in terms of expressions of personal beliefs about group characteristics—and stereotype awareness—in terms of perceived societal beliefs about the groups. This differentiation is highly relevant, because only expressions of personally endorsed group stereotypes can be unambiguously interpreted in line with SJT as indicators of internalized ingroup inferiority, whereas expressions of societal group stereotypes may also be interpreted as perceptions of social reality and predominant societal beliefs and evaluations, with which the participants may or may not agree. Such expressions would be rather in line with conceptualizations of perceived prejudice and stigmatization of the RIM. In this line, we additionally coded perceived group evaluations that occurred without specific trait characteristics. We summarize these two variables as ‘perceived stigmatization’. Finally, we also analyzed responses with regard to expressions of societal and systemic issues that (dis)advantage one or the other group as explanation for status differences.

The current analyzes were run with data from four studies originally conducted to investigate predictors of group evaluations (i.e., ingroup vs. outgroup favoritism) across four samples of disadvantaged groups. The original goal of these studies was to investigate whether and to what extent individual differences and group differences in system-justifying beliefs, conservatism, and social dominance orientation were related to group evaluations (these pre-registered analyzes will be reported elsewhere). We had specifically chosen to invite members of these four disadvantaged groups because previous research (Essien et al., 2021) let us expect that these groups would vary markedly with regard to group evaluations, with gay and lesbian participants showing ingroup favoritism, overweight and elder participants showing outgroup favoritism, and Black and African American participants’ attitudes to be located somewhere in between.

The main objective of the current analyzes is to provide an overview over the relative frequencies of each response category for each sample and compare these with previous experimental findings (Jost, 2001). Because the studies contained a number of further measures, we are also able to report results of additional exploratory analyzes. First, we report the average values of direct and indirect measures of group preferences and ingroup evaluation for the four samples testing whether they replicate response patterns observed in Essien et al. (2021). Second, we explored relationships between participants’ status explanations and group evaluations. Given that SJT (Jost and Banaji, 1994; Jost, 2020) conceptualizes both, group stereotyping and group evaluations, as potential manifestations of system-justifying tendencies, one may expect a positive relationship between the two. Third, we explored the relationships between participants’ (open-ended) status explanations with participants’ individual system-justifying tendencies and three measures assessing different aspects of conservative ideology. We conducted these analyzes because based on Jost’s reasoning, one may expect that system-justifying patterns of group stereotyping (i.e., outgroup-favorable, ingroup-unfavorable) in those participants that express higher levels of system justifying tendencies, higher social dominance orientations and more conservative political ideologies. Status attributions to perceived stigmatization and societal issues on the other hand, should be less likely in participants with higher levels of system justifying tendencies, social dominance orientation and/or political conservatism (as recently argued in Howard et al., 2021). Finally, we explored the relationship between participants’ group status explanations and two measures of experienced discrimination and stigma consciousness.

Note that the current research is exploratory in nature: Although we had preregistered all measures included in this data collection with the Open Science Framework^2^, neither the current hypotheses nor the reported analyzes were pre-registered. We report how we determined our sample size, all data exclusions, and all measures in the study. We did not conduct any experimental manipulations (Simmons et al., 2012).

## Materials and Methods

### Sample Size Determination

We had planned and pre-registered the current data collection with the aim to conduct correlational analyzes. Therefore, we aimed at providing sufficient test power (1-β = 0.80 at α < 0.05) to test for the small effect sizes of *r* = 0.20, thus requiring valid data of 191 participants per study. With potential data exclusions in mind (see pre-registration), we slightly overpowered all four studies.

Participants were recruited using Prolific^3^ and received 2.88 GBP for a study duration of 20–23 min. For all samples, we applied prescreening criteria based on country of residence (United States), native language (English), and no participation in any of the other or previous studies of our lab. A further selection criterion was related to social group membership: For Sample 1, we only invited Prolific users who had registered their sexual orientation with Prolific as gay or lesbian; for Sample 2, we only invited Prolific users who had registered as Black or African American; for Sample 3 we only invited Prolific users with a self-reported Body Mass Index higher than 35 and for Sample 4 we only invited Prolific users aged between 60 and 75 years.

Study 1 was initially commenced by 222 Prolific users, Study 2 by 230, Study 3 by 228 and Study 4 by 223 users. Eventually, *n_1_* = 210, *n_2_* = 215, *n_3_* = 219, *n_4_* = 214 participants completed the data collection and provided informed consent for data storage and analysis after completion (see section “Procedures”). Following our pre-registered exclusion criteria, we excluded the data of 16 participants (*n_1_* = 4, *n_2_* = 2, *n_3_* = 5, *n_4_* = 8) who indicated no nationality or a nationality other than the United States, and 19 participants who did not self-categorize as belonging to the specific disadvantaged groups in question (*n_1_* = 2, *n_2_* = 4, *n_3_* = 13). Note that we also excluded data of one participant from Study 3 who self-categorized as “slightly overweight” but whose BMI of 20.5 fell into the normal-weight category of the WHO and one participant from Study 4 whose age was below the inclusion criteria of 60 years.

We had also pre-registered to exclude data of participants who failed an attention check twice (*n*_1_ = 7, *n*_2_ = 10, *n*_3_ = 3, *n_4_* = 3). Because exclusion of these participants did not alter results in any meaningful way, we decided to deviate from our preregistration and include data of these participants (see also Supplementary Material).

All studies were approved by the ethics committee of the Psychology Department at Universität Hamburg (AZ 2020_311).

### Participants

The current analyzes rely on valid data of 819 participants from four samples.

#### Gay and Lesbian Participants

Analyzes are based on data of *N* = 205 persons who self-identified as homosexual (99 female, 87 male, and 18 diverse or non-binary). The majority of participants, *n* = 148 (72.2%), self-categorized as White, further 19 (9.3%) participants self-categorized as Black or African American, 16 (7.8%) as Asian, 13 (6.3%) as Hispanic or Latinx, one (0.5%) as Native Hawaiian or Pacific Islander, and 8 (3.9%) as other. Participants’ age ranged from 18 to 73 years (*Md* = 29, *M* = 30.56, *SD* = 10.6).

#### Black and African American Participants

Analyzes are based on data of *N* = 209 persons who self-categorized as Black or African American (113 female, 96 male). Participants’ age ranged from 18 to 76 years (*Md* = 31, *M* = 32.23, *SD* = 9.65).

#### Overweight Participants^4^

Analyzes are based on data of *N* = 200 participants who self-categorized as overweight (123 female, 68 male, 9 diverse or non-binary). The majority of participants, *n* = 164 (82%), self-categorized as White, further 17 (8.5%) self-categorized as Hispanic or Latinx, 6 (3%) as Black, 6 (3%) as Asian, 2 (1.5%) as Native Hawaiian or Pacific Islander, and 5 (2.5%) as other. Participants’ age ranged from 18 to 73 years (*Md* = 36, *M* = 38, *SD* = 12.3). Participants’ BMI ranged between 25.45 and 78.56 (*M* = 43.85, *SD* = 9.44). According to self-reports, 16 participants (8%) self-categorized as slightly overweight, 85 (42.5%) as moderately overweight, and 99 (49.5%) as extremely overweight^5^.

#### Older Participants

Analyzes are based on data of *N* = 205 persons aged between 60 and 75 years (*Md* = 64, *M* = 65.36, *SD* = 4.2; 126 female, 79 male). The majority of participants, *n* = 189 (92.2%), self-categorized as White, further four (2%) participants self-categorized as Black or African American, four (2%) as Hispanic or Latinx, two (1%) as Asian, one (0.5%) as Native Hawaiian or Pacific Islander, and 2 (1%) as other.

### Measures

Note that the central measure of group status perception and explanation was embedded into a survey containing a number of further measures. We shortly list all measures in the section “Procedure,” and describe in detail those that we use for current analyzes. All materials, raw data, and analysis scripts can be found in OSF^2^.

#### Status Measures

Participants completed a two-item adaptation of the MacArthur Scale of Subjective Social Status (Adler et al., 2000) to measure perceived status positions of social groups in society. We presented a ladder with 10 rungs, ranging from 1 (*lowest status*) to 10 (*highest status*) along with an item asking participants where they thought {lesbian or gay people, Black people, overweight people, older people} in the US stood on this ladder in general and where they thought {straight people, White people, normal-weight people, younger people} in the US stood on this ladder in general. Note that in our item formulation we only used the group labels without explicitly referring to them as ingroups or outgroups nor as disadvantaged or advantaged groups (see Materials in OSF).

Directly afterward, we presented the status value that participants had chosen for their ingroup and outgroup and asked them for an explanation of their response. Specifically, instructions read: “You have indicated a rank of {value} for {ingroup/outgroup} people in the US. What do you think are the reasons for this social ranking? Please list *all* the reasons that you can spontaneously think of. It is enough to list keywords, you do not need to give elaborate explanations.” Participants provided their responses in an unlimited multi-line text box. Participants always first completed the ingroup status explanation followed by the outgroup status explanation.

#### Group Attitude Measures

For comparison to previous work, we used the direct and indirect measure typically employed by project implicit (e.g., Essien et al., 2021).

##### Implicit Association Test

We had created four parallel versions of the Implicit Association Test (IAT, Greenwald et al., 1998) for a previous research project (Degner and Essien, unpublished manuscript) adapted for use in the Qualtrics survey software (Carpenter et al., 2019).^6^ In each IAT, participants categorized attribute words according to their meaning as *Good* vs. *Bad* and target images as belonging to one of the target categories. In Sample 1, the target categories were *Gay vs. Straight*, each represented by 10 images of same-gender and different-gender couples in romantic, yet non-sexual poses (e.g., holding hands, hugging, kissing) from a commercial photo stock website^7^. In Sample 2, the target categories were *Black people vs. White people*, each represented by ten portrait pictures selected from the Chicago Face Database (CFD; Ma et al., 2015). In Sample 3, the target categories were *Overweight persons vs. Normal-weight persons*, each represented by ten morph images, each created from three individuals from Google image searches. In Sample 4, the target categories were *old persons vs. young persons*, represented by ten portrait images, of which two were selected from the Chicago Face Database (Ma et al., 2015) and 18 where AI-generated portraits created with an online service (Generated Photos, 2021). Because copyrights apply to some of these images, we are not at liberty to provide open access to all materials. We provide an overview of all stimuli in OSF and are committed to sharing materials with other researchers upon personal request.

We used the analysis tool provided by iatgen (Carpenter et al., 2019) to calculate an IAT D score (the D600 algorithm, Greenwald et al., 2003), coded such that scores above zero indicate faster responses when the ingroup targets and good attributes vs. outgroup targets and bad attributes shared a response key than when the key assignment was the opposite, which is typically interpreted as indicator of relative ingroup preference. Therefore, we excluded IAT-data of eight participants (*n_1_* = 1, *n*_2_ = 6, *n*_3_ = 1) with ≥10% of trials with response times ≤ 300 ms (excessive speed criterion, see Greenwald et al., 2003). All IATs were characterized by satisfying reliability indices with α_1_ = 0.871, α_2_ = 0.883, α_3_ = 0.841, α_4_ = 0.825.

##### One-item Preference Measure

For reasons of comparability with available research in this domain (e.g., Essien et al., 2021), we employed a one-item group preference measure with a 7-point scale, ranging from outgroup preference (i.e., *I strongly prefer Straight/White/normal-weight/young people to Gay/Black/Overweight/old people*) via no preference (i.e., *I like Gay/Black/Overweight/old people and Straight/White/normal-weight/old people equally*) to ingroup preference (i.e., *I strongly prefer Gay/Black/Overweight/old people to Straight/White/normal-weight/young people).*

##### Ingroup Evaluation Measure

As further measure of ingroup evaluation, we employed five items adapted from Luhtanen and Crocker (1992), which measure positive identification with the ingroup (i.e. “*I am proud to be Gay/Black/Overweight/old*.”, “*I prefer Gay/Black/Overweight/old people to Straight/White/normal-weight people.”, “I regret being Gay/Black/Overweight/old* (r).”, “*I am glad to be Gay/Black/Overweight/old*.”, “*I feel good about being Gay/Black/Overweight/old*.”). The scale was characterized by satisfying reliability indices with α_1_ = 0.813, α_2_ = 0.798, α_3_ = 0.828, α_4_ = 0.796.

##### Ingroup Identification Centrality

We adapted two items from Leach et al. (2008; “*Being Gay/Black/Overweight/old is an important part of my identity.”, “Being Gay/Black/Overweight/old is an important part of how I see myself.”*) with satisfying reliability indices with α_1_ = 0.915, α_2_ = 0.940, α_3_ = 0.823, α_4_ = 0.895.

#### Ideology

We employed the eight-item System Justification Scale (Jost and Thompson, 2000; Kay and Jost, 2003) which measures perceptions of the fairness, legitimacy, and justifiability of the prevailing social system, and showed satisfying reliability indices with α_1_ = 0.872, α_2_ = 0.890, α_3_ = 0.862, α_4_ = 0.781. In order to capture the different facets of conservative beliefs (cf. Jost et al., 2003), we employed the 11-item of the Resistance to Change-Beliefs Scale (RC-B; White et al., 2020), which measures individual beliefs concerning the desirability of change versus stability, in terms of preference for tradition and preference for gradual change. The scale showed satisfying reliability indices with α_1_ = 0.860, α_2_ = 0.765, α_3_ = 0.877, α_4_ = 0.890. Secondly, we employed the 16-item Social Dominance Orientation Scale, which measures general beliefs that hierarchies in society are inevitable and natural, and respective support for group-based dominance and opposition to equality (Jost and Thompson, 2000). This scale showed satisfying reliability indices with α_1_ = 0.917, α_2_ = 0.909, α_3_ = 0.901, α_4_ = 0.909. In all these measures, responses were collected on 7-point Likert-like scales ranging from 1 (*strongly disagree*) to 7 (*strongly agree*). Finally, we asked participants to indicate their general political views and ideology on a single item with a 7-point scale ranging from *extremely liberal* to *extremely conservative*.

#### Stigma Consciousness and Experienced Discrimination

##### Stigma Consciousness

We adapted the ten-item Stigma Consciousness Questionnaire (SCQ; Pinel, 1999), which measures to what extent participants expect to be stereotyped by others based on their sexual orientation, racialized group membership, or weight status (e.g., “*When interacting with {Straight, White, normal-weight, young} people, I feel like they interpret all my behaviors in terms of the fact that I am {Gay, Black, overweight, old}*.”, “*Stereotypes about {Gay, Black, overweight, old} people have not affected me personally.*”). Responses were collected on a seven-point Likert-like scale ranging from “*strongly disagree*” to “*strongly agree*”. Scale reliabilities were satisfying with α_1_ = 0.820, α_2_ = 0.836, α_3_ = 0.868, α_4_ = 0.810.

##### Experienced Discrimination

We selected six items from the Daily Discrimination subscale (Williams et al., 1997) that measure experiences of unfair treatment in daily live *(“You are treated with less courtesy than other people.”, “You are treated with less respect than other people.”, “You receive poorer service than other people at restaurants or stores.”, “People act as if they think you are not as good as they are.”, “You are called names or insulted.”, “You are threatened or harassed.”).* Responses were collected on a five-point Likert-like scale with the anchors “never,” “rarely,” “sometimes,” “often,” “always.” Scale reliabilities were satisfying with α_1_ = 0.898, α_2_ = 0.913, α_3_ = 0.918, α_4_ = 0.913.

### Procedure

Data collection was conducted online in March 2021 using Qualtrics^8^ for creating and running the survey and Prolific^3^ for participant recruitment. Upon recruitment, participants were informed that the general goal of the study was to investigate social group attitudes and their relations to group identification. The study started with a welcome page that contained an initial attention check (Oppenheimer et al., 2009) requiring participants to click on a logo instead of the continue button. If participants failed the attention check, they received a notice and were asked to re-read instructions on the welcome page.

Data collection then began with the two group attitude measures: the evaluative IAT immediately followed by the one-item group preference measure. Next, participants were asked to indicate demographic information, including self-categorizations with regard to the social category in question, thus their sexual orientation (Study 1), ethnicity (Study 2), weight-status (Study 3) and/or age (Study 4). All following measures were only presented to those participants, who fulfilled the studies’ inclusion criteria, thus self-categorized as homosexual or gay (Study 1), Black or African American (Study 2), at least slightly overweight (Study 3), or indicated their age to be equal or above 60. Participants who did not fulfill these inclusion criteria were forwarded to an end-of-survey message and their data discarded from analyzes. The remaining survey began with the ingroup identification evaluation measure (adopted from Luhtanen and Crocker, 1992) and the ingroup identification centrality items (adapted from Leach et al., 2008). Items of both scales were presented together in an individually randomized sequence. The survey then continued with the System Justification scale (Jost and Thompson, 2000), followed by the two group status items and assessments of status explanations in an open-ended response format. Afterward, participants completed additional ideology measures, including exploratory measures of perceived status stability, perceived group entitativity, and perceived group permeability. Finally, participants completed the experienced discrimination scale and a stigma consciousness questionnaire. The survey ended with an adapted measure of ingroup and outgroup friendship orientations.

After completion of all measures, participants received detailed information on the background and hypotheses of the current research and were asked to confirm their initial consent for storage, analyses, and open accessibility of their (anonymous) data, and were rerouted back to Prolific where they received payment independent of consent. A complete copy of the surveys including all measures is available in the OSF^2^.

### Data Preparation

In order to analyze participants’ open-ended responses explaining ingroup and outgroup status, we developed a coding system that expanded the coding employed by Jost (2001). Like Jost (2001) we first coded whether participants’ open responses contained stereotype-related references to the ingroup or the outgroup and whether these implied a positive, negative, or neutral/ambivalent evaluation. Expanding Jost’s system, we further categorized each statement as indicating either stereotype endorsement versus stereotype awareness^9^ and also coded perceptions of generalized group evaluations. Additionally, we coded whether systemic aspects were provided as explanations of ingroup and outgroup status. We provide a short summary description of the coding system here and further detailed information and coding exemplars in the Supplementary Figure S1 and Supplementary Table S1.

Stereotype endorsement captured statements expressing a personal belief or conviction with regard to characteristics of the ingroup or the outgroup. This applied to statements about group characterizations or characterizations of individual group members (e.g., “*Blacks are …*”, see examples in Supplementary Table S1). We used a very inclusive coding approach. That is, whenever participants’ merely mentioned trait words without any further specification (e.g., “*laziness*”), we interpreted these as group-trait associations and coded them as expressions of stereotype endorsement.

Perceived stigmatization contains two sub-facets: stereotype awareness and perceived evaluation by others. Stereotype awareness captures expressions of perceptions of others’ beliefs or convictions with regard to individual traits and group characteristics, signaled by verbal markers such as “*(they/we) are viewed as*,” “*(they/we) are assumed to be*,” “*People believe that (they/we) are…*” (see Supplementary Table S1). Perceived evaluations refer to expressions of perceived generalized evaluations of the ingroup or the outgroup. We included into this category expressions of perceived general (dis)like, (dis)respect, (dis)approval, acceptance or rejection, admiration or hatred, as well as explicit references to terms like bias, stigma, or prejudice (e.g., “*Overweight people are shamed and stereotyped. They are looked down upon. They are openly ridiculed. They are mocked. They are not taken seriously*.”; see Supplementary Table S1 for examples).

Systemic aspects were defined as any mentions of perceived societal, institutional, or organizational advantages or disadvantages of either the ingroup or the outgroup (e.g., “*Black peoples’ struggles,” “white privilege”*). When possible, we further coded responses as referring to economic status or access to resources (e.g., *“wealth gap,” “poverty”*), access to status-related opportunities such as employment (e.g., “s*ystemic racism denying access to jobs and resources*”), education (e.g., “*There is deliberate zoning to keep Black children out of better schools*”), housing (e.g., “*ability to obtain housing and business loans*”) or health care (e.g., “*lack of healthcare and appropriate sex education*”), societal norms and normative fit (e.g., “*it is like the world is made for skinny people*”), disparities in legal status (e.g., “*straight people have more legal protections*”), or any acts of discrimination (e.g., “*racist practices still around*”) or lack of discrimination (e.g., “*Literally no one will ever discriminate a person for being straight.*”). In each case, we coded whether the disparity was referred to as an ingroup (dis)advantage and/or an outgroup (dis)advantage (see examples in Supplementary Table S1).

We additionally coded whether participants mentioned tradition or historical roots of systemic aspects (e.g., “*Even since the end of slavery, the government set up ways that African Americans cannot succeed or be successful”*) and the perceived stability, malleability or inescapability of the system (e.g., “*Gay people have come a long way in the last 50 years but things could and should be better*,” “*Black people are the recipients of systematic racism, which means it is inescapable*.”).

Responses of all participants of Samples 1–3 were coded independently by the first and second author of this paper and all cases of disagreement were resolved via discussion, responses of participants from Sample 4 were only coded by the second author of the paper.

## Results and Discussion

### Overview

We first report results of the status perception and group evaluation measures in order to verify that status perceptions indeed support the assumption that participants perceive their ingroup as lower in status than a majority outgroup. We report the descriptive statistics of all further measures in the Supplementary Table S2. We then report in detail the results of frequency analyses of participants’ open-ended responses explaining group status. Finally, we report explored interrelations between open-ended responses and group attitude measures, experienced discrimination, and stigma consciousness.

#### Status Perceptions

Our analyses are based on the assumption that participants perceive the status of their ingroup as relatively lower compared to the status of the respective comparison outgroup. In order to verify whether this was indeed the case, we inserted participants’ status perceptions into a two (Status group: ingroup vs. outgroup) by four (participant group: Gay vs. Black vs. Overweight vs. Older Aged) ANOVA with repeated measure on the first factor. The analysis revealed a significant main effect of status group, *F*(1,815) = 816.994, *p* < 0.001, η^2^_*p*_ = 0.501, 95% *CI* = [0.456,0.540], a significant main effect of participants group *F*(3,815) = 70.505, *p* < 0.001, η^2^_*p*_ = 0.206, 95% *CI* = [0.158,0.251], and a significant interaction effect, *F*(3,815) = 77.239, *p* < 0.001, η^2^_*p*_ = 0.221, 95% *CI* = [0.172,0.267]. As depicted in Table 1, participants in Samples 1–3 perceived the societal status of their ingroups as significantly lower relative to the status of the respective comparison outgroup, and this effect was strongest in the sample of Black participants. These results replicate findings with members of these three minority groups (Degner and Essien, unpublished manuscript) indicating that, based on the self-perception of participants, it is adequate to label these groups as disadvantaged groups. Participants in Sample 4, however, did not show a consistent status difference perception. Specifically, the number of participants aged 60–75 who perceived ingroup status to be lower than outgroup status (*n* = 98) was almost equal to the number of participants to who perceived ingroup status to be higher than outgroup status (*n* = 89), and a few participants (*n* = 18) indicated no status difference. Thus, based on participants self-perceptions, there was no agreement on whether people aged 60–75 associate being older with lower status.

**TABLE 1:** Group status perceptions.

	Perceived ingroup status		Perceived outgroup status		*t*-Test (within)
	*M*	(*SD*)	*M*	(*SD*)	
Study 1	4.620	(1.408)	7.727	(1.753)	*t*(204) = −18.940, *p* < 0.001, *d* = −1.492, 95%*CI* [1.273, 1.711]
Study 2	4.852	(1.741)	8.263	(1.665)	*t*(208) = −20.018, *p* < 0.001, *d* = −1.355, 95%*CI* [1.142, 1.567]
Study 3	4.005	(1.297)	6.620	(1.462)	*t*(199) = −19.160, *p* < 0.001, *d* = −1.443, 95%*CI* [1.223, 1.663]
Study 4	5.200	(1.613)	5.444	(1.675)	*t*(204) = −1.353, *p* = 0.178, *d* = −0.094, 95%*CI* [−0.099, 0.288]

*d(repeated measures), see Lenhard and Lenhard (2016).*

The Online Supplement reports descriptive analyses of all further quantitative measures (Supplementary Table S3), which replicated previous findings and rankings of groups in terms of intergroup attitudes (e.g., Essien et al., 2021). Importantly, these supplemental analyses demonstrate remarkable levels of heterogeneity in terms of ingroup and/or outgroup favoritism: Gay and lesbian participants exhibited consistent ingroup favoritism in the IAT and self-report measures; Black and African American participants exhibited outgroup favoritism in the IAT but ingroup favoritism and ingroup pride in self-report measures; older participants exhibited outgroup favoritism in the IAT but no group preference in self-report measures; and overweight participants exhibited outgroup favoritism in both the IAT and the self-report measure.

#### Status Explanations

##### Stereotype Endorsement

Overall, stereotype endorsement was lower than observed in Jost (2001), but varied strongly between groups. Specifically, 37 (18.0%) gay and lesbian participants, 38 (18.2%) Black participants, 88 (43.8%) overweight participants, and 150 (73.2%) older participants expressed *any* type of ingroup or outgroup stereotype in their open responses. Table 2 lists the proportion of participants whose stereotype endorsements contained positive versus negative characterizations of ingroup versus outgroup.

**TABLE 2:** Frequency of endorsed stereotypes (percentages in parentheses).

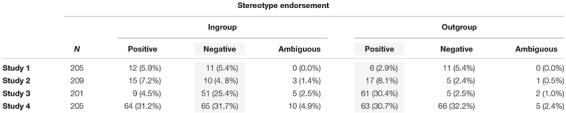

For further analyses, we collapsed response frequencies across system-justifying group stereotypes (i.e., unfavorable ingroup and/or favorable outgroup, marked in light gray in Table 2) and opposing group stereotypes (i.e., favorable ingroup and/or unfavorable outgroup). We then conducted a McNemar-test, a non-parametric test comparing frequencies of these two forms of stereotype endorsement as within-participant variables. There was no significant difference within the sample of gay and lesbian participants (14 vs. 21; χ^2^ = 1.241, *p* = 0.265), African American participants (23 vs. 18; χ^2^ = 0.593, *p* = 0.441), and older participants (90 vs. 91; χ^2^ = 0.000, *p* = 1.000), only in the sample of overweight participants did more participants express system-justifying stereotypes than opposite stereotypes (78 vs. 13; χ^2^ = 54.613, *p* < 0.001).

In summary, while only very few lesbian and gay and Black participants explained group status with stereotypic characteristics of group members, group characteristics were more frequently invoked by overweight and older participants. Importantly, in Samples 1, 2, and 4, negative ingroup characterizations and positive outgroup characterizations were not more frequent than other group characterizations. Only the stereotype endorsement of 25–30% of overweight participants appeared to fit the pattern predicted by SJT. Further content analyses of stereotype endorsement in the sample of overweight and elderly participants indicated that their high levels of stereotype endorsement were partly due to frequent discussion of health issues related to being overweight and aging (see Supplementary Table S3).

##### Perceived Stigmatization

Overall, the proportion of participants who mentioned any kind of stereotype awareness or perceived evaluation was higher as compared to stereotype endorsements in the first three samples, with 128 (62.4%) gay and lesbian participants, 55 (26.32%) African American participants, 120 (59.70%) of overweight participants. Among older participants perceived stigmatization was mentioned by 76 participants (37.1%), and thus less frequent than stereotype endorsement. As can be seen in Table 3, the proportion of participants mentioning perceived evaluations is relatively high, whereas stereotype awareness is generally less frequent. Again, we inspected whether the relative frequency of system-legitimizing stereotypes or evaluations (ingroup-negative and/or outgroup-positive, columns marked in gray in Table 3) was different from opposite stereotypes or evaluations. This was indeed the case. That is, the number of participants expressing perceived stereotyping to be system-legitimizing (ingroup-negative and/or outgroup-positive) was significantly higher than the number of participants expressing opposed perceived stereotyping, with 21 vs. 2; *p* < 0.001 in the sample of gay and lesbian participants, 10 vs. 0; *p* = 0.002 in the sample of Black participants, 53 vs. 2; *p* < 0.001 in the sample of overweight participants, and 15 vs. 3; *p* = 0.002 in the sample of older participants.

**TABLE 3:** Frequency of perceived stereotyping and group evaluations.

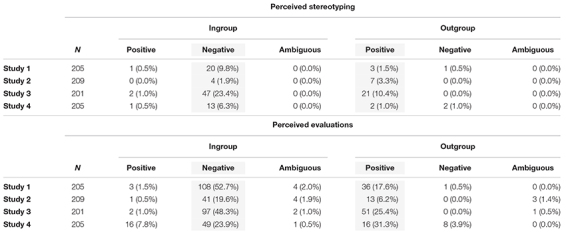

Perceived group evaluations was generally more frequently expressed and showed a similar pattern: A higher number of participants reported perceived negative ingroup evaluations and/or positive outgroup evaluations than vice versa, with 117 vs. 4; χ^2^ = 103.669, *p* < 0.001 in the sample of gay and lesbian participants, 46 vs. 1; χ^2^ = 41.191, *p* < 0.001 in the sample of Black participants, 104 vs. 2; χ^2^ = 96.236, *p* < 0.001 in the sample of overweight participants, and 55 vs. 18; χ^2^ = 18.254, *p* < 0.001, in the sample of elderly participants.

##### Systemic Reasoning

In a final set of analyses, we explored the frequency of participants whose responses included references to systemic reasons for ingroup and/or outgroup status. This was the case for a majority of participants in each sample. Specifically, 187 (91.2%) participants in the gay and lesbian sample, 181 (89.6%) participants in the Black sample, 136 (67.7%) participants in the overweight sample, and 161 (78.5%) participants in the elderly sample mentioned systemic explanations of group status. Whenever possible, we further coded whether their reasoning referred to perceived ingroup (dis)advantages and/or perceived outgroup (dis)advantages. In samples 1–3, a visibly larger group of participants described ingroup disadvantages and outgroup advantages than vice versa (see Table 4), whereas older participants of Sample 4 mentioned both systemic advantages and disadvantage for both ingroup and outgroup.

**TABLE 4:** Systemic reasoning.

		Systemic reasoning			
		Ingroup		Outgroup	
	*N*	Advantage	Disadvantage	Advantage	Disadvantage
**Study 1**	205	12 (5.9%)	132 (64.4%)	145 (70.7%)	1 (0.5%)
**Study 2**	209	3 (1.4%)	153 (73.2%)	159 (76.1%)	1 (0.5%)
**Study 3**	201	6 (3.0%)	99 (49.3%)	115 (57.2%)	0 (0.0%)
**Study 4**	205	61 (29.8%)	80 (39.0%)	64 (31.2%)	60 (29.3%)

We identified a number of recurring themes that we coded as systemic explanations of group statuses (see Table 5), most importantly the relative (dis)advantage with regard to material resources (e.g., poverty, lack of generational wealth), opportunities (e.g., access to work, housing, education, health care), and experiences of discrimination.

**TABLE 5:** Content analysis of systemic status explanations.

		Systemic reasoning II								
		Material resources		Opportunities		Discrimination		Other mentions		
	*N*	Ingroup	Outgroup	Ingroup	Outgroup	Ingroup	No outgroup	Legal	Historical	Normative
		advantage	advantage	advantage	advantage	discrimination	discrimination*	status	roots^#^	fit
**Study 1**	205	10 (4.9%)	13 (5.9)	4 (1.5%)	57 (27.8%)	102 (49.8%)	55 (26.8%)	50 (24.4%)	39 (19.0%)	112 (54.6%)
**Study 2**	209	1 (1.0%)	88 (42.1%)	1 (0.5%)	102 (48.8%)	112 (53.6%)	21 (10.0%)	17 (8.1%)	36 (17.2%)	17 (8.1%)
**Study 3**	201	2 (1.0%)	31 (15.4%)	1 (0.5%)	62 (30.8%)	48 (23.9%)	19 (9.5%)	2 (1.0%)	0 (0.0%)	51 (25.4%)
**Study 4**	205	61 (29.8%)	59 (28.8%)	19 (9.3%)	44 (21.5%)	14 (6.8%)	3 (1.5%)	0 (0.0%)	7 (3.4%)	18(8.8%)

*Absence of discrimination explicitly mentioned as outgroup advantage/outgroup privilege; ^#^mentioned mostly with reference to religious norms in Sample 1 and to slavery in Sample 2.

### Relations of Status Explanations With Intergroup Attitudes, Ideology, Experienced Discrimination and Stigma

In the following analyses, we explored whether coded status explanations extracted from participants’ open-ended responses were related to participants’ intergroup attitudes, ideological beliefs, or interindividual differences in experienced discrimination and stigma consciousness. Because our coding procedure resulted in binary variables (i.e., we coded whether a specific content was either mentioned or not), we conducted simple independent group comparisons between those participants who mentioned any of the aforementioned contents in their group status explanations and those who did not. Table 6 reports the effect sizes Cohen’s *d* of these exploratory independent *t*-tests for each of the three group attitude measures, the ideology measures as well as the two measures of stigma consciousness and experienced discrimination, respectively.

**TABLE 6:** Group comparisons reported as Cohen’s *d* [95% CI].

	Stereotype endorsement				Perceived stigmatization				Systemic reasoning			

	**Sample 1**	**Sample 2**	**Sample 3**	**Sample 4**	**Sample 1**	**Sample 2**	**Sample 3**	**Sample 4**	**Sample 1**	**Sample 2**	**Sample 3**	**Sample 4**
	**(37 vs. 167)**	**(38 vs. 170)**	**(87 vs. 112)**	**(150 vs. 54)**	**(128 vs. 76)**	**(55 vs. 153)**	**(120 vs. 79)**	**(76 vs. 128)**	**(187 vs. 17)**	**(181 vs. 27)**	**(135 vs. 64)**	**(150 vs. 54)**
**Evaluation IAT**	0.375 [0.017, 0.733]	0.138 [-0.219, 0.495]	0.084 [-0.196, 0.365]	0.120 [-0.431, 0.191]	-0.407 [-0.694, -0.121]	-0.334 [-0.649, -0.018]	-0.400 [-0.687, -0.112]	0.043 [-0.240, 0.327]	0.013 [-0.448, -0.509]	-0.151 [-0.562, 0.260]	-0.157 [-0.455, 0.141]	0.007 [-0.329, 0.344]
**One-item evaluation**	0.167 [-0.189, 0.524]	0.196 [−0.156, 0.549]	−0.002 [−0.282, 0.278]	−0.155 [−0.466, 0.157]	−0.414 [−0.700, −0.127]	−0.236 [−0.544, 0.074]	−0.295 [−0.581, −0.010]	−0.213 [−0.498, 0.071]	−0.038 [−0.535, 0.458]	−0.383 [−0.789, 0.023]	0.021 [−0.277, 0.318]	0.010 [−0.34, 0.327]
**Ingroup pride**	0.326 [−0.032, 0.683]	0.375 [0.021, 0.728]	0.083 [−0.197, 0.363]	−0.322 [−0.635, −0.009]	−0.237 [−0.521, 0.048]	0.031 [−0.277, 0.339]	0.029 [−0.255, 0.313]	0.111 [−0.173, 0.395]	−0.169 [−0.666, 0.328]	0.068 [−0.337, 0.442]	−0.176 [−0.474, 0.122]	0.063 [−0.274, 0.399]
**Ingroup identity**	0.035 [−0.321, 0.391]	0.497 [0.142, 0.852]	0.052 [−0.228, 0.332]	−0.242 [−0.554, 0.069]	−0.385 [−0.672, −0.099]	0.188 [−0.121, 0.496]	−0.191 [−0.475, 0.094]	0.010 [−0.274, 0.294]	−0.369 [−0.866, 0.129]	0.194 [−0.210, 0.599]	−0.331 [−0.630, −0.032]	−0.072 [−0.408, 0.265]
**SJ beliefs**	−0.296 [−0.653, 0.062]	−0.099 [−0.451, 0.253]	−0.142 [−0.423, 0.138]	−0.252 [−0.564, 0.060]	0.468 [0.180, 0.755]	0.285 [−0.024, 0.595]	0.510 [0.222, 0.799]	0.144 [−0.140, 0.429]	0.698 [0.197, 1.199]	0.497 [0.090, 0.904]	0.251 [−0.047, 0.550]	0.158 [−0.179, 0.494]
**SDO**	−0.062 [−0.418, 0.294]	−0.674 [−1.032, −0.317]	−0.119 [−0.399, 0.161]	−0.055 [−0.366, 0.256]	0.606 [0.316, 0.896]	0.342 [0.032, 0.652]	0.560 [0.271, 0.849]	0.211 [−0.074, 0.495]	0.520 [0.021, 1.019]	1.019 [0.603, 1.435]	0.343 [0.044, 0.643]	0.210 [−0.127, 0.547]
**Change resistance**	−0.025 [−0.381, 0.332]	−0.099 [−0.451, 0.253]	−0.165 [−0.492, 0.070]	−0.089 [−0.400, 0.222]	0.486 [0.180, 0.755]	0.146 [−0.162, 0.455]	0.508 [0.220, 0.796]	0.114 [−0.170, 0.398]	0.671 [0171, 1.172]	0.495 [0.088, 0.902]	0.493 [0.192, 0.794]	0.267 [−0.070, 0.605]
**Political ideology**	−0.115 [−0.472, 0.241]	−0.119 [−0.471, 0.233]	−0.195 [−0.476, 0.085]	−0.087 [−0.399, 0.224]	0.299 [0.013, 0.584]	−0.008 [−0.316, 0.300]	0.278 [−0.007, 0.563]	0.017 [−0.266, 0.301]	0.449 [−0.050, 0.947]	0.164 [−0.240, 0.569]	0.278 [−0.021, 0.577]	0.299 [−0.039, 0.637]
**Experienced discrimination**	0.198 [−0.158, 0.555]	0.148 [−0.204, 0.500]	0.016 [−0.265, 0.296]	0.221 [−0.091, 0.533]	−0.023 [−0.307, 0.261]	−0.044 [−0.352, 0.264]	−0.434 [−0.722, −0.147]	0.097 [−0.187, 0.381]	0.080 [−0.416, 0.577]	−0.078 [−0.482, 0.327]	−0.354 [−0.654, −0.055]	0.131 [−0.206, 0.468]
**Stigma consciousness**	0.147 [−0.209, 0.504]	0.240 [−0.113, 0.592]	0.037 [−0.243, 0.318]	0.074 [−0.237, 0.386]	−0.389 [−0.675, −0.103]	−0.260 [−0.569, 0.049]	−0.844 [−1.140, −0.548]	−0.258 [−0.543, 0.027]	−0.286 [−0.783, 0.211]	−0.746 [−1.156, −0.335]	−0.457 [−0.758, −0.157]	0.033 [−0.303, 0.370]

*Effect size calculations were adjusted for different group sizes by including weights for groups sizes into the calculation of the pooled standard deviation (see Hedges and Olkin, 1985), Positive d-values imply that those who did not mention a response category (column) scored higher in the DV (line). Negative d-values indicate that those who mentioned a response category (column) scored higher in the DV (line) than those who do not.*

As can be seen from the most left panel of Table 6, there appear to be no systematic differences in any of the dependent variables between those participants who expressed (any kind of) stereotype endorsement and those who did not. It thus seems that the endorsement of group stereotypes in general was not related to intergroup attitudes, suggesting that stereotype endorsement and outgroup favoritism were independent from each other. Also, participants who expressed group stereotypes as explanations of group status differences were not characterized by higher levels of system-justifying and conservative beliefs.^10^

There were, however, a number of systematic group differences with regard to the perceived stigmatization response category: Participants whose responses contained references to stereotype awareness and/or perceived evaluation tended to exhibited higher IAT scores and higher levels of self-reported group preferences (both indicating higher ingroup favoritism or lower outgroup favoritism, respectively) than those who did not mention these issues (see Table 6, central panel). There were also differences in the ideology measures: Participants who mentioned perceived stigmatization as explanations for group status expressed significantly lower levels of system-justifying beliefs, social dominance orientation, and traditionalist resistance to change, although these effects appeared to be smaller in the sample of African American and older participants as compared to the two other samples of disadvantaged group members. These results appear consistent with previous findings that system justifying mindsets were related to reduced recollections of perceived discrimination (e.g., Owuamalam et al., 2017).

Additionally, we observed systematic relationships to stigma consciousness: Participants who reported perceived stigmatization were characterized by higher averaged levels of stigma consciousness in all four samples, which lends validity to our coding of the open responses. The relationship to experienced discrimination was less consistent and only significant in Sample 3.

Finally, systemic reasoning for group status and status differences was similarly related to ideological variables. That is, participants who mentioned societal and/or systemic reasons for ingroup or outgroup status, expressed significantly lower system-justifying beliefs, lower social dominance orientation, and lower traditionalist resistance to societal change than those who did not mention any systemic reasons. Again, we observed a positive relationship with stigma consciousness in three out of four samples, with those expressing systemic reasons for group status differences being characterized by higher levels of stigma consciousness. Again, we observed no systematic relationship with experienced discrimination, nor with group attitudes.

Overall, the relations between the ideological variables and our coding of participants’ open-ended responses lend validity to our findings and indirectly fit SJT: Participants who referred to perceived stigmatization and/or provided systemic reasons for group status differences, were on average more liberal, less likely to perceive the current system as fair and legitimate, and had lower levels of social dominance orientation and resistance to change (cf. Howard et al., 2021).

Our exploratory analyses of interrelations between participants responses and other available measures revealed some unexpected results. Most striking to us were the interrelations with the group attitude measures. Specifically, IAT scores and the one-item preference measures were only related to perceived stigmatization and no other response code, a result confirmed by multiple regression analyzes reported in the Supplementary Table S4. Importantly, we observed a *reversed* relationship, that is participants who reported perceived stigmatization as explanations for group status were more likely to exhibit ingroup favoritism (Sample 1) or reduced outgroup favoritism (Samples 2 and 3) than those who did not mention perceived stigmatization. Only in Sample 4 did we not observe any such interrelations (see Supplementary Table S4).

## General Discussion

The current research explored whether and to what extent members of disadvantaged groups express group stereotypes as explanations of perceived ingroup and outgroup status or status differences. Our content analyses of open-ended responses of over 800 participants from four different disadvantaged groups revealed that only few participants referred to stereotypical group characterizations as explanations for ingroup or outgroup status. Moreover, we observed substantial variability between groups, with overweight and older participants more often using stereotypical group characterizations, but gay and lesbian or Black and African American participants rarely using stereotypical group characterizations. Importantly, among participants who mentioned stereotypic group characterizations, system legitimizing characterizations (of negative ingroup traits and/or positive outgroup traits) were not more frequent than other characterizations. Instead, participants in all four samples frequently referred to perceived stigmatization (such as awareness of others’ group stereotypes and perceived group evaluations) and systemic aspects to explain ingroup disadvantage.

These findings diverge from previous experimental studies, in which a majority of participants expressed system-justifying ingroup and/or outgroup stereotypes (Jost and Burgess, 2000; Jost, 2001). These findings are, however, in line with the shared reality explanation of stereotype expressions pointed out by Rubin and Hewstone (2004) and illustrate the importance of a theoretical separation of measures of stereotype awareness versus stereotype endorsement. Realizing societal views about one’s ingroup and understanding these as reasons for the ingroup status does not necessarily imply endorsement of these views as personal beliefs (see also consideration on passive reflection of reality in Owuamalam et al., 2018b). Also, contrary to assumptions in SJT, participants, who did mention group stereotypes in their status explanations were not characterized by higher levels of system-justification tendencies, or other ideological beliefs. There were also no systematic relationships between group attitudes and stereotype endorsement: Participants who expressed stereotype endorsement did not systematically differ in their IAT scores or their self-reported ingroup or outgroup favoritism from those who did not express any stereotype endorsement. This is somewhat inconsistent with SJT, which construes both variables as interrelated manifestations of system-justification tendencies in members of disadvantaged groups.

In our view, there are two potential reasons why our results diverge from previous findings from Jost’s (2001) study. First, it is possible that coding procedures in previous research may have conflated stereotype endorsement with stereotype awareness and perceived stigmatization and may thus have overestimated the degree of stereotype internalization. We do, however, believe that this clear conceptual separation is paramount: Expressions of stereotype awareness and perceived stigmatization cannot be interpreted as indicators of stereotype endorsement and internalized personal beliefs as hypothesized in SJT. Second, group status and status differences were operationalized very differently in past and present research. Present findings relied on data collected from members of real-world groups, thus from people who have potentially have made long-term—often life-long—experiences of pervasive disadvantages and stigmatizations. Previous studies, on the other hand, were conducted with students from prestigious universities and relied on temporal manipulations of status perceptions (i.e., by providing information of relatively higher or lower socioeconomic success of graduates from their own or another university; Jost and Burgess, 2000; Jost, 2001). Although experimental manipulations do provide advantages for causal interpretation, they may suffer from threats to external validity with regard to real-world phenomena and risk mischaracterizing or even trivializing real-live group disparities and inequality (e.g., Jost, 2019).

Previous experimental studies are also open to alternative interpretations. For example, one could speculate whether the obserevd high levels of stereotype use (Jost, 2001) primarily reflected attributional strategies that people use when explaining relatively lower levels of *advantage* rather than relatively higher levels of *dis*advantage. Alternatively, one could speculate whether stereotype use is only a spontaneous initial strategy that people use when first learning about an ingroup disadvantage (and when they have little or no experience of belonging to a systematically disadvantaged social group), but that they may abandon in the long. Such reasoning is supported by recent developmental research indicating that young children initially and dominantly use personal attributions of novel group status disparities but shift toward structural explanations during middle childhood (Peretz-Lange et al., 2021). Such differences in initial versus long-term use of stereotype-based versus structural attributions might account for differences between present findings and previous research.

The present findings appear consistent with assumptions of the Rejection Identification Model (RIM; Branscombe et al., 1999) in that many participants expressed perceived stigmatization as well as systemic factors as causes of status differences. Note, however, that relationships of these responses with social identification were not entirely consistent with the theory: Whereas gay and lesbian participants (Sample 1) and overweight participants (Sample 3) who mentioned perceived stigmatization and/or systemic aspects exhibited higher levels of ingroup identification, which is consistent with the RIM, no such relationships were observed among Black and African American participants (Sample 2). Conversely, we observed that the few Black and African American participants who mentioned group stereotypes as status explanations were characterized by lower levels of ingroup identification, which again can be considered as consistent with the RIM, but this pattern was not observed in the other two samples. Note, however, that we had only employed a two-item measure of identity centrality and may have missed other important facets of social identity (Leach et al., 2008). Furthermore, ingroup identification was skewed toward relatively high identification in Samples 1 and 2 and toward relatively low identification in Samples 3 and 4 (see Supplementary Table S2). At this current point in time, we cannot say whether these variance restrictions are a result of unrepresentative sampling or should be considered a valid characterization of these groups. Correlational analyses thus need to be interpreted with caution.

Taken together, our current exploratory analyses provide only limited support for SJTs assumption of stereotype use as attributions of ingroup status in members of disadvantaged groups. That is not to say that members of disadvantaged groups may not employ group stereotypes at all. In the present studies, only a minority of participants used group stereotypes when asked to explain abstract *group* status. But they may nevertheless use stereotypes when making sense of their own or other’s concrete and *individual* experiences or outcomes. For example, research on race-status associations suggests that stereotyping might differ depending on whether they are assessed at the individual (i.e., exemplar) level or group level (Dupree et al., 2021).

Note, however, that the mere observation of group stereotypes being expressed as explanations of ingroup status in members of disadvantaged groups would actually not provide sufficient empirical support for the claim that stereotype use stems from an underlying system justification motive. For example, the recently proposed social identity model of system attitudes (SIMSA, Owuamalam et al., 2018a,b) disputes the existence of a unique system-level motive and instead argues that instances of outgroup favoritism and system legitimization can be more parsimoniously explained by social identity considerations. Specifically, they show that identification with a social system along with a hope or expectation for future system change and ingroup advancement is also related to system-justifying attitude expressions (e.g., Owuamalam et al., 2021). Other theoretical approaches also would predict stereotype use in members of disadvantaged groups without relying on an underlying system justification motive. For example, the recently proposed Bias of Crowds model (BoC; Payne et al., 2017) conceptualizes measures of intergroup attitudes as indicators of properties of places and situations. The BoC account proposes that context-based availability and accessibility of group stereotypes and prejudice can influence individual thinking and behavior (e.g., Stelter et al., 2021). Given that members of disadvantaged groups—like all members of society—are continuously exposed to societally shared stereotypes and prejudice, one may expect effects of context-based stereotype activation and application among members of disadvantaged groups (cf. Essien et al., 2021). This approach does not rely on the assumption of personally endorsed or internalized stereotypes, because it locates the activation of stereotypes in societal contexts rather than in the individual, an assumption which is also consistent with the shared reality explanation (Rubin and Hewstone, 2004) and the passive reflection assumption (Owuamalam et al., 2018b) in social identity-based approaches. These considerations indicate that further theorizing and research is needed to better understand the use of group stereotypes in members of disadvantaged groups.

Finally, one repeated pattern of results should be highlighted, namely the relatively consistent relationships between participants group attitudes and the different indicators of perceived stigmatization. We observed in three out of four samples that participants whose status explanations contained references to perceived stigmatization exhibited higher levels of ingroup favoritism (in Sample 1) or lower levels of outgroup favoritism (in Samples 2 and 3) on both direct and indirect attitude measures. Multiple regression analyses (see Supplementary Materials) confirmed that perceived stigmatization was the only predictor of intergroup attitudes. Further relationships with the stigma consciousness questionnaire and the experienced discrimination scale yielded widely consistent results. Only in the sample of older participants did we not observe such interindividual differences and relations. These results point toward an intriguing dissociation of group-level vs. individual-level effects. On the group level, our results are consistent with previous findings showing that group attitudes in disadvantaged groups closely align with societal stigma (Essien et al., 2021). We had selected the four disadvantaged groups for this study because they are associated with different levels of societal stigma and their mean group attitudes indeed closely follow this ranking: The sample of gay and lesbian participants exhibited ingroup favoritism, the sample of overweight and older participants exhibited outgroup preference and the sample of Black and African Americans ranked in between. At the individual level, however, we mostly observed *reversed* relationships between group attitudes and indicators of stigma: Participants who demonstrated higher levels of stigma consciousness or experienced discrimination and participants who reported perceived stigmatization as status explanations, exhibited higher levels of ingroup favoritism (Sample 1) or lower levels of outgroup favoritism (Samples 2 and 3). Before jumping to conclusions, these unexpected findings should be replicated, ideally in a pre-registered, hypotheses-testing approach. Should these patterns be replicable, they would point toward intriguing questions of which group- and individual-level processes in members of disadvantaged groups are responsible for groups (on average) to align their group attitudes with societal stigmatization but individual members of these groups with high levels of stigma consciousness diverge from societal stigmatization. The differences between samples with regard to effect size and direction of these relationships also point toward potentially important characteristics of different disadvantaged groups that warrant further attention.

To conclude, we believe that our exploratory analyses do not only challenge the generalizability of previous findings of stereotype use in members of disadvantaged groups, they also point toward novel and intriguing questions that should be addressed in future research.

## Data Availability Statement

The datasets presented in this study can be found in the Open Science Framework; https://osf.io/2ey4q/.

## Ethics Statement

The studies involving human participants were reviewed and approved by Local Ethics Committee Faculty of Psychology and Human Movement Science Von-Melle-Park 5 20146 Hamburg. The participants provided their written informed consent to participate in this study.

## Author Contributions

JD conceived of the presented idea, programmed the studies, and supervised data collection as part of several Masters theses at Universit t Hamburg. J-CF and JD developed the coding manual, conducted the response coding, and statistical analyses. All authors discussed the results and contributed to the final manuscript.

## Conflict of Interest

The authors declare that the research was conducted in the absence of any commercial or financial relationships that could be construed as a potential conflict of interest.

## Publisher’s Note

All claims expressed in this article are solely those of the authors and do not necessarily represent those of their affiliated organizations, or those of the publisher, the editors and the reviewers. Any product that may be evaluated in this article, or claim that may be made by its manufacturer, is not guaranteed or endorsed by the publisher.

## Footnotes

1 In the other two studies, participants completed trait ratings of the groups on status-relevant (intelligent, hard-working, skilled) and status irrelevant attributes (friendly, honest, interesting, see Jost and Burgess, 2000), but these were not elicited as explanations of status differences.

2
https://osf.io/2ey4q/

3
https://www.prolific.co/

4 The survey contained a note to participants explaining that we used the terms normal-weight and overweight as reference to two social categories because they related to the widely used classification system by the WHO, that we are aware that these are very diverse groups and also that there are many different forms and causes of overweight, and that we did not imply that being overweight was in any way a negative defect a person has.

5 When applying the criteria of the World Health Organization, 7 participants (3.5%) would be categorized as pre-obese, 27 (13.5%) as obese class I, 39 (19.5%) as obese class II, and 125 (62.5) as obese class III, respectively (with *n* = 2 not diagnosable because they did not report their body height and/or weight).

6
http://iatgen.org/

7
shotshop.com

8
www.qualtrics.com

9 Note that we had further coded whether the following five sub-facets were captured by expressions of stereotypes: competence, warmth, ideology, physical health and physical appearance. Because of the low frequencies, we refrained from analyzing these facets. Interested readers can find a short description of these facets, and all coded data in the OSF^2^.

10 A very similar pattern of results was observed when reducing this analysis to mentions of only SJT-compatible stereotype endorsement (ingroup-negative, outgroup positive).
