# Supplementary material for: Do Members of Disadvantaged Groups Explain Group Status With Group Stereotypes?
Source: Front Psychol. 2021 Nov 18;12:750606. doi: 10.3389/fpsyg.2021.750606 (PMC8636313; doi:10.3389/fpsyg.2021.750606)
Supplement: Supplementary file 1 [file Data_Sheet_1.pdf]

Do Members of Disadvantaged Groups Explain Group Status with Group Stereotypes?

Supplemental Materials

Juliane Degner[1]

Joelle-Cathrin Floether[1]

Iniobong Essien[2]

[1]Universität Hamburg, Germany

[2]FernUniversität in Hagen, Hagen, Germany

Figure S.1 *Schematic Summary of the Coding Scheme*

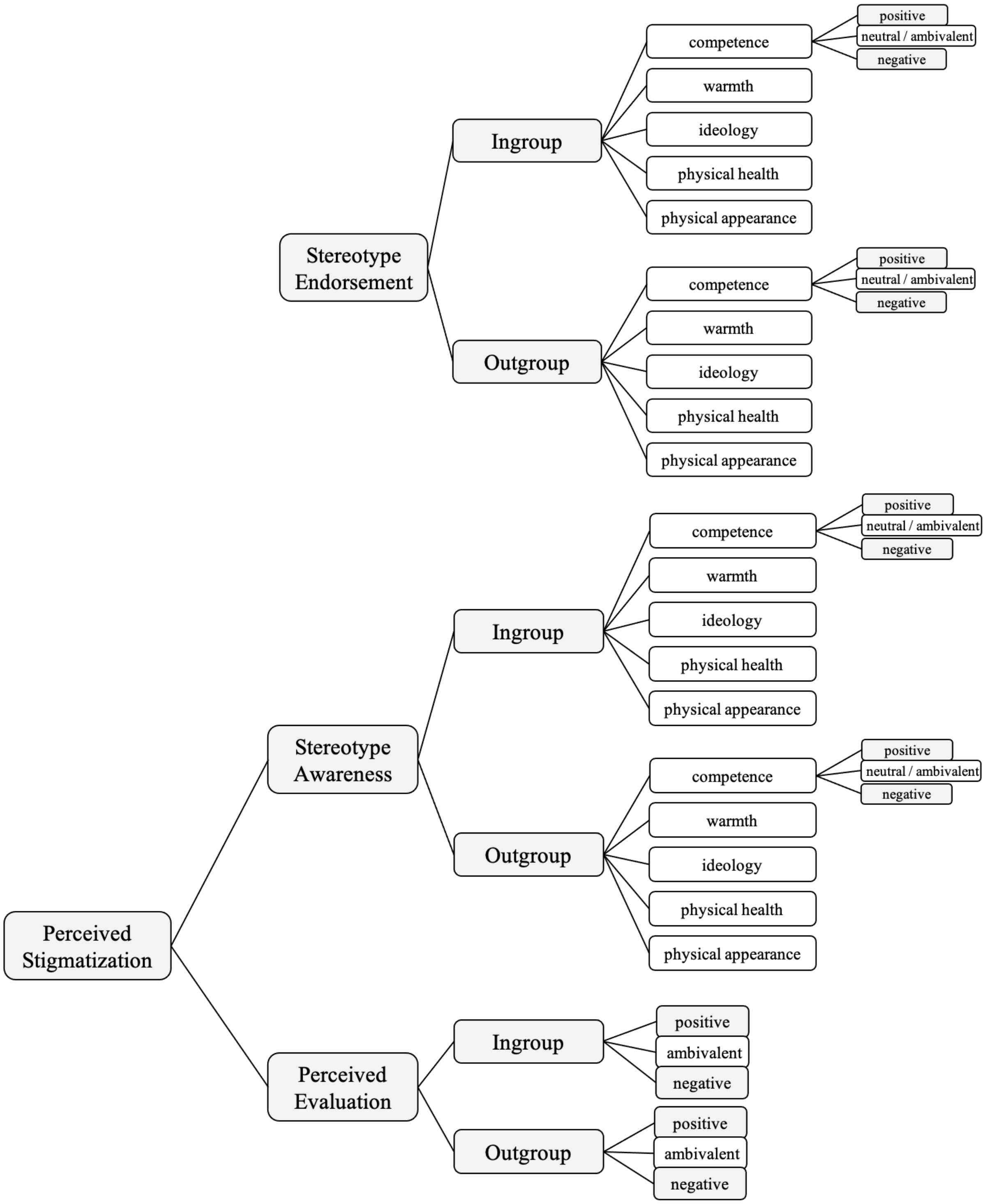

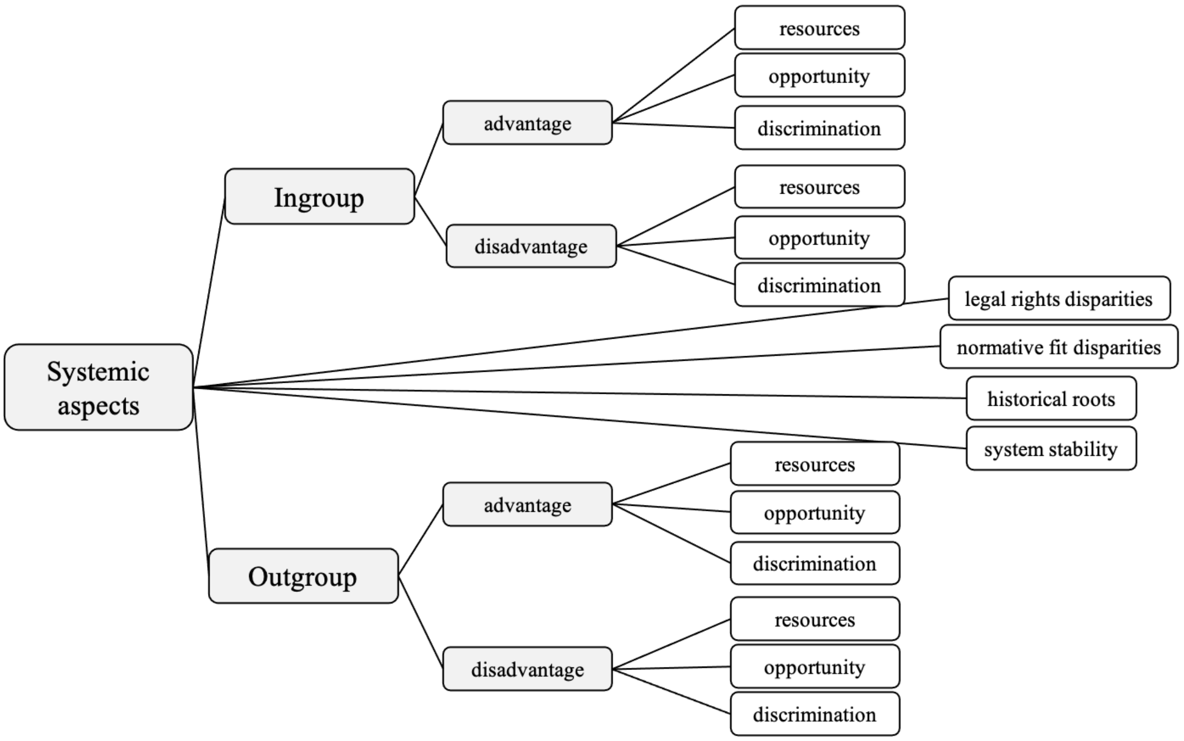

Note: Categories presented in white rectangles were not included into analyses, for two reasons: In the case of specific stereotype content in the stereotype endorsement and stereotype awareness categories cell frequencies were extremely low and not comparable between groups.

In the case of specific systemic issues of group dis/advantages, categories cannot be considered as disjunct (i.e., the categories tend to overlap considerably, resulting in lowered reliability of coding).

Table S.1. Exemplar Responses Illustrating the Coding Scheme

	**STEREOTYPE ENDORSEMENT**	
	**ingroup**	**outgroup**
**Sample 1**	*unorthodox*	*straights often very judgmental*
**Sample 2**	*not willing to work harder, laziness*	*white people are more educated, (...) extremely smart*
**Sample 3**	*low mobility, low motivation, lack of intelligence*	*smarter, more ambitious, go getter, not lazy*
**Sample 4**	*more medical problems, illness cognitive decline wisdom, respect*	*I feel that they are smart, quick witted and know how to manipulate the system to their advantage. Young people have the ability, but most are too impatient to carry through.*
	**STEREOTYPE AWARENESS**	
	**ingroup**	**outgroup**
**Sample 1**	*a lot of people believe that we are twisted or immoral*	*not seen as gross, not seen as sexual*
**Sample 2**	*Blacks are viewed as being less smart than whites*	*people find them trustworthy*
**Sample 3**	*people tend to judge overweight people as lazy, undisciplined and stupid*	*they are seen as healthy*
**Sample 4**	*the general idea that old people are cranky and not willing to learn new things*	*People feel they are smarter and better able to accomplish things.*
	**PERCEIVED EVALUATION**	
	**ingroup**	**outgroup**
**Sample 1**	*unaccepted, homophobia, stereotypic view,*	*socially accepted*
**Sample 2**	*we are not respected in America*	*societal status is high, many people like white people*
**Sample 3**	*overweight people are shamed and stereotyped. They are looked down upon. They are openly ridiculed. They are mocked. Medical professionals won't take them seriously*	*normal weight people are often glorified*
**Sample 4**	*Disrespect, Sometimes old people are disregarded in this country*	*Our society worships youth. Younger people seem to be more widely accepted.*

	**SYSTEMIC REASONS**	
	**ingroup**	**outgroup**
**systemic (dis)advantage**	*Black peoples' struggles*	*white privilege, had time to accumulate social standing*
**(lack of) access to resources**	*wealth gap, economic growth, low income, most of them have worked all their lives and saved up money for retirement*	*Most healthy weight people have more time and money to spend on being healthy*
**(lack of) availability of opportunities**	*lack of safe healthcare, education, media, community, workplaces, places to worship, places to drink*	*they run the country, they have all the power, Youth have more opportunities. Opportunities such as education and government help.*
**acts/lack of discrimination**	*There is a lot of racial discrimination that still goes on against Black people today. Whether if it just racist people or systemic racism it affects people greatly*	*not ever having had to or will ever experience racism*
**disparities in legal status**	*Gay marriage was only recently legalized and there are still many laws that allow states to discriminate against gay people, straight people have more legal protection*	
**disparities in normative fit**	*straight people are literally considered the norm for society and its considered unique or strange to be LGTB+. Everything is catered with the idea of being straight Standard. Normal. Fit in. Things are designed for them. Beauty.*	
**tradition or historical roots**	*Slavery, It was not that long ago, when being black was a literal death sentence, everything in the US and its history has been oriented with straight people in mind*	
**perceived stability, malleability of system**	*although it is getting slightly better, it is still not great, Black people are being heard now more than before, It has been like this since the beginning of time*	

Further Descriptive Analyses

Table S.2. Descriptive Analyses of Quantitative Measures

	Study1		Study 2		Study 3		Study 4	
	M (*SD*)	*d*	*M* (*SD*)	*d*	*M* (*SD*)	*d*	*M* (*SD*)	*d*
IAT [a]	0.166 (0.466)	0.357	-0.208 (0.426)	-0.494	-0.403 (0.467)	-0.403	-0.473 (0.408)	-1.158
One-Item Preference [b]	5.283 (1.248)	1.028	4.938 (1.279)	0.733	3.690 (1.371)	-0.301	4.283 (1.033)	-0.274
Ingroup Pride [b]	5.540 (1.059)	1.450	5.851 (1.000)	1.851	2.063 (0.966)	-2.006	4.187 (1.121)	-0.167
Identity centrality [b]	5.393 (1.422)	0.979	5.723 (1.535)	1.122	2.760 (1.677)	-0.740	3.945 (1.596)	-0.032
System Justification[b]	2.730 (1.113)	-1.141	3.522 (1.306)	-0.366	2.911 (1.193)	-0.913	3.678 (1.030)	-0.312
Social Dominance[b]	1.627 (0.640)	-2.143	1.825 (0.728)	-1.615	1.689 (0.656)	-1.998	1.771 (0.707)	-3.153
Change Resistance[b]	2.396 (0.676)	-0.894	2.929 (0.627)	-0.114	2.656 (0.735)	-0.468	3.092 (0.755)	0.122
Political Ideology [b]	3.080 (2.617)	-0.352	4.140 (2.429)	0.058	3.600 (2.458)	-0.163	4.200 (2.286)	0.085
Experienced Discrimination[c]	2.197 (0.772)	1.550	2.297 (0.866)	1.304	2.478 (0.860)	1.719	1.666 (0.651)	1.023
Stigma Consciousness [b]	4.532 (0.990)	0.537	4.526 (1.144)	0.460	4.633 (1.143)	0.553	3.824 (0.917)	-0.192

Note. Effect size estimates refer [a] to the comparison against zero for the IAT, [b] to the comparison against the midpoint of the scales representing neither agreement or disagreement, and [c] to the comparison against 1 representing absence of experienced discrimination.

Table S.3. Absolute Frequencies (and percentages) of Stereotype Endorsement Categories in Sample 3 (Overweight Participants) and Sample 4 (older participants)

	Ingroup				Outgroup			
**Sample3**	negative	positive	ambiguous	no mention	negative	positive	ambiguous	no mention
Competence	28 (13.9%)	8 (4.0%)	1 (0.5%)	163 (81.1%)	2 (1.0%)	37 (18.4%)	0 (0%)	161 (80.1%)
Warmth	0 (0%)	5 (2.5%)	0 (0%)	195 (97.0%)	3 (1.5%)	3 (1.5%)	0 (0%)	194 (96.5%)
Ideology[a]	0 (0%)	0 (0%)	0 (0%)	200 (99.5%)	0 (0%)	0 (0%)	0 (0%)	200 (99.5%)
Physical health	33 (16.4%)	0 (0%)	3 (1.5%)	164 (81.6%)	1 (0.5%)	26 (12.9%)	1 (0.5%)	172 (85.6%)
Physical appearance	5 (2.5%)	0 (0%)	0 (0%)	195 (97.0%)	0 (0%)	21 (10.4%)	0 (0%)	179 (89.1%)
**Sample4**	**Ingroup**				**Outgroup**			
Competence	22 (10.7%)	52 (25.4%)	0 (0%)	204 (63.4%)	37 (18.0%)	43 (21.0%)	1 (0.5%)	123 (60.0%)
Warmth	11 (5.4%)	16 (7.8%)	1 (0.5%)	176 (85.9%)	15 (7.3%)	8 (3.9%)	0 (0%)	181 (88.3%)
Ideology[a]	6 (2.9%)	0 (0%)	2 (1.0%)	197 (96.1%)	0 (0%)	4 (2.0%)	0 (0%)	200 (97.6%)
Physical health	40 (19.5%)	1 (0.5%)	5 (2.4%)	158 (77.1%)	3 (1.5%)	15 (7.3%)	2 (1.0%)	184 (89.8%)
Physical appearance	5 (2.4%)	0 (0%)	2 (1.0%)	197 (96.1%)	0 (0%)	10 (4.9%)	1 (0.5%)	193 (94.1%)

Note. Cells with grey shading can be regarded as system-justifying stereotype contents, negative value imply conservative characterizations, positive values imply progressive characterizations

Auxiliary Analyses

Multiple regression of group attitude variables.

To further corroborate interrelationships between the group attitude measures and the open-ended responses (see Table 6 in the main manuscript), we conducted multiple regression analyzes, using the group attitude measure (i.e., IAT score, one-item measure) as dependent variable, and stereotype endorsement, perceived stereotyping, and systemic rationalization as binary simultaneous predictor variables. Results of Samples 1, 2, and 3 indicated that perceived stigmatization was the only and independent predictor of the IAT score and the self-report item, whereas group attitudes in Sample 4 of older participants were unrelated to status explanations.

Table S.4. Multiple regression analyses with IATs-scores as DV

	**Sample 1**	**Sample 2**	**Sample 3**	**Sample 4**
**IAT**	R = .236, F(3, 200) = 3.933, p = .009	R = .155, F(3, 198) = 1.618, p = .187	R = .200, F(3, 194) = 2.696, p = .047	R = .060, F(3, 200) = 0.240, p = .868
ß(endorse)	-.133 (.056)	-.041(.570)	-.030 (.673)	.056 (.429)
ß(stigma)	.188 (.007)	.141 (.048)	.184 (.010)	-.028 (.697)
ß(system)	-.021 (.757)	.023 (.755)	.045 (.529)	.001 (.993)
	**Sample 1**	**Sample 2**	**Sample 3**	**Sample 4**
**Self-report Preference**	R = .204, F(3, 200) = 2.905, p = .036	R = .162, F(3, 204) = 1.829, p = .143	R = .147, F(3, 195) = 1.437, p = .233	R = .117, F(3, 200) = 0.924, p = .430
ß(endorse)	-.053 (.443)	-.047 (.506)	.007 (.919)	.058 (.418)
ß(stigma)	.195 (.006)	.087 (.214)	.148 (.040)	.095 (.179)
ß(system)	-.008 (.906)	.106 (.140)	-.031 (.665)	.006 (.929)

Correlations between group attitude measures, stigma consciousness, and perceived discrimination.

To further validate the observed interrelationships between mentions of perceived stigmatization and intergroup attitudes reported in Table 6, we further explored the relationship of the group attitudes measures with the stigma consciousness questionnaire and the experienced discrimination measure. These analyses yielded partly consistent correlations: Participants with higher levels of stigma consciousness exhibited higher levels of ingroup favoritism (Sample 1) and lower levels of outgroup favoritism (Samples 2, 3, 4) in IATs and self-report measures. Correlations were numerically similar but smaller and mostly non-significant for the ingroup pride measure, but reversed in the sample of older participants. Correlations with experienced discrimination were mostly the same in direction, but less consistent across samples and significance. Significant correlations were observed only with the self-reported group preferences, not with IATs or ingroup pride.

Table S.5. Correlations between group attitude measures and stigma consciousness and experienced discrimination (*p*-values in parentheses)

	**Stigma Consciousness**				**Experienced Discrimination**			
	**Sample 1**	**Sample 2**	**Sample 3**	**Sample 4**	**Sample 1**	**Sample 2**	**Sample 3**	**Sample 4**
Evaluation	.192	.128	.155	.053	-.070	.139	.061	-.012
IAT	(.006)	(.068)	(.029)	(.448)	(.315)	(.047)	(.395)	(.867)
One-Item	.401	.230	.193	.174	.246	.121	.217	.082
Evaluation	(.000)	(001)	(.006)	(.013)	(.000)	(.081)	(.002)	(.244)
Ingroup	.167	.107	.063	-.240	-.005	.005	.079	-.217
Pride	(.017)	(.121)	(.375)	(.001)	(.948)	(.937)	(.269)	(.002)
